# *HoxBlinc*: a key driver of chromatin dynamics in NUP98 fusion–driven leukemia

**DOI:** 10.1172/JCI191355

**Published:** 2025-04-01

**Authors:** Jian Xu, Wei Du

**Affiliations:** 1 Division of Hematology and Oncology, University of Pittsburgh School of Medicine, Pittsburgh, Philadelphia, USA.; 2 UPMC Hillman Cancer Center, Pittsburgh, Pennsylvania, USA.

## Abstract

Nucleoporin 98 (*NUP98*) fusion oncogenes are known to promote aggressive pediatric leukemia by disrupting chromatin structure and modulating the expression of homeobox (*HOX*) genes, yet the precise molecular events are unclear. In this issue of the *JCI*, K. Hamamoto et al. explore the mechanistic underpinnings of NUP98 fusion–driven pediatric leukemia, with a focus on aberrant activation of the *Hoxb*-associated long, noncoding RNA (lncRNA) *HoxBlinc*. The authors provide compelling evidence that *HoxBlinc* plays a central role in the oncogenic transformation associated with NUP98 fusion protein. The study underscores a CTCF-independent role of *HoxBlinc* in the regulation of topologically associated domains (TADs) and chromatin accessibility, which has not been fully appreciated in previous research on the *NUP98* fusion oncogenes. The discovery of *HoxBlinc* lncRNA as a downstream regulator of NUP98 fusion oncoproteins offers a potential target for therapeutic intervention in pediatric leukemia.

## Nucleoporin 98 fusion–driven leukemia and therapeutic challenge

Nucleoporin 98 fusion–driven (NUP98 fusion–driven) leukemia represents a distinct and challenging category of hematological malignancies (1, 2). This condition arises when the *NUP98* gene on chromosome 11 fuses with a variety of partner genes, oftentimes homeobox transcription factors (such as HOXA9, HOXD13, plant homeodomain finger 23 [PHF23], or HHEX), or components of epigenetic machinery (including KDM5A, NSD1, or BPTF). This fusion results in the production of abnormal proteins that disrupt normal hematopoiesis and contribute to leukemia development. It probably does so by altering the expression of genes involved in hematopoietic stem and progenitor cell (HSPC) differentiation and self-renewal, ultimately resulting in leukemic transformation (3).

Leukemia driven by NUP98 fusions is often characterized by aggressive clinical behavior (2). These leukemias tend to have a poor prognosis due to their resistance to conventional therapies, at least in part because of widespread epigenetic changes resulting from its fusion partners, such as NSD1 or KDM5A, which alter gene expression and promote resistance to standard treatment (3, 4). Topologically associated domains (TADs) help ensure that enhancers interact with their target promoters while insulating nontarget genes from activation (5, 6, 7). Disruption of TADs is now considered an oncogenic driver. NUP98 fusion proteins can reorganize TADs, thereby altering chromatin interactions and enabling persistent activation of oncogenic pathways, which may contribute to therapy resistance.

## The HOXB gene cluster long, noncoding RNA *HoxBlinc*

*HoxBlinc*, previously linked to early hematopoiesis, has been identified as a carcinogenic long noncoding RNA (lncRNA) that plays a crucial role in NPM1-mutant (NPM1c^+^) acute myeloid leukemia (AML) through interaction with and recruitment of its partner mixed-lineage leukemia protein 1 (MLL1) to the promoter regions of NPM1c^+^-specific genes, fostering H3K4me3 modifications and thereby activating gene transcription (8). Overexpression of *HoxBlinc* in mouse models enhances the self-renewal capacity of hematopoietic stem cells (HSCs), leading to the development of an AML-like disease. Similar to NPM1c^+^, NUP98 fusions aberrantly activate both *HOXA* and *HOXB* genes, facilitating leukemogenesis (9, 10). However, the mechanism by which *HoxBlinc* regulates *NUP98* fusion–driven leukemia remains unclear.

**Figure 1:**
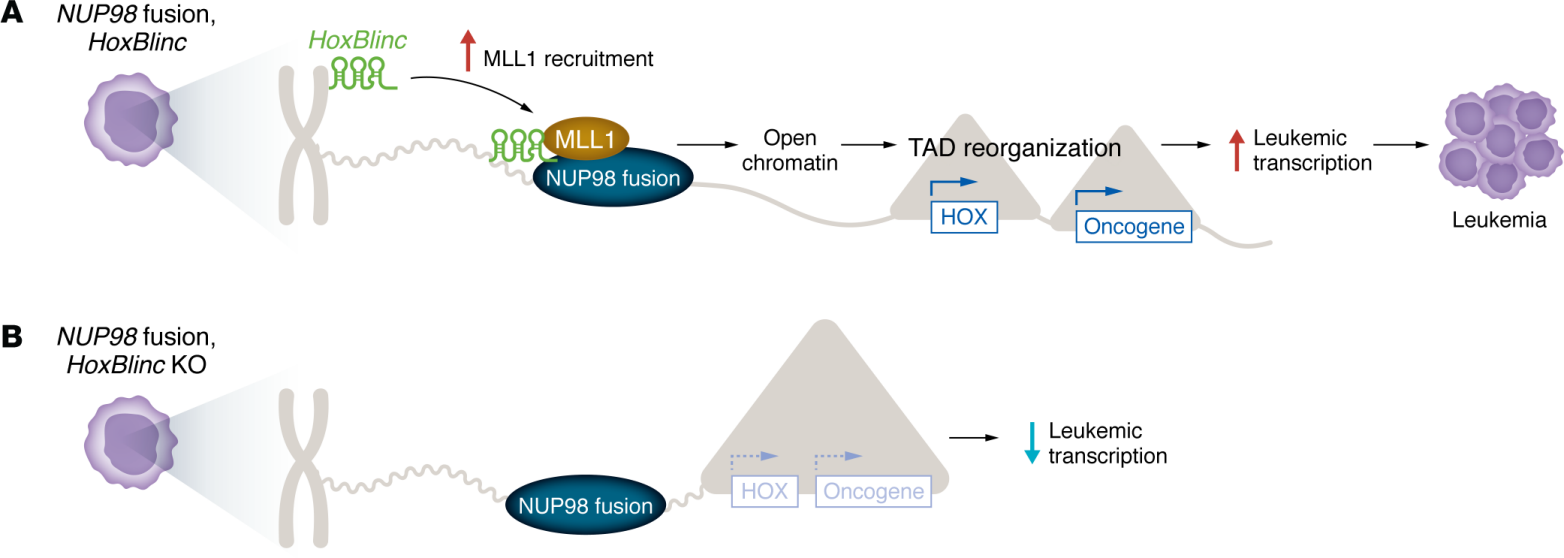
*HoxBlinc* modulates oncogenic transcription and leukemogenesis in *NUP98* fusion–driven leukemia. (**A**) *NUP98* fusion induces aberrant activation of *HoxBlinc*, causing a reorganization of TADs that alters chromatin interactions. *HoxBlinc* facilitates chromatin accessibility of MLL1 at promoter regions, ultimately enhancing the expression of HOX and other oncogenic genes. (**B**) Loss of *HoxBlinc* reduces MLL1 recruitment and decreases leukemic gene transcription.

A particularly intriguing finding of Hamamoto et al. in this issue of the *JCI* is the identification of the role of the lncRNA *HoxBlinc* in *NUP98* fusion–driven leukemogenesis (11). The authors found that *HoxBlinc* lncRNA was aberrantly activated in 961C B cell acute lymphoblastic leukemia (B-ALL) cells in comparison with control BaF3 B cells. Using chromatin isolation by RNA purification sequencing (ChIRP-Seq) and the assay for transposase-accessible chromatin using sequencing (ATAC-Seq), they demonstrated that *HoxBlinc* bound to specific promoter regions of *Hoxb* and *Hoxa* loci, which are involved in transcriptional regulation. *HoxBlinc* binding was associated with increased chromatin accessibility and transcriptional activation of target genes in 961C cells compared with control BaF3 B cells. Additionally, Hi-C analysis revealed alterations in TADs in 961C cells, with 97 new TADs formed and 56 TADs decreased, many of which were enriched in genes involved in transcription regulation and leukemogenesis, suggesting a crucial role of *HoxBlinc* in mediating chromatin remodeling and promoting the activation of leukemic signatures in NUP98-PHF23–driven leukemia (Figure 1) (11).

To investigate the role of *HoxBlinc* lncRNA in NUP98-PHF23 fusion oncoprotein–driven homeotic gene expression and leukemogenesis, Hamamoto and co-authors knocked out *HoxBlinc* in two NUP98-PHF23 transformed B-ALL cells (961C and A1929). The KO resulted in a reduction of *HoxBlinc* transcripts and a decrease in the expression of homeotic oncogenes such as *Hoxa*, *Hoxb*, and *Meis1*. Gene set enrichment analysis (GSEA) revealed that *HoxBlinc* is crucial for the NUP98-HOXA9 fusion signature and pathways associated with cell differentiation, immune response, and hematopoiesis. Further experiments showed that loss of *HoxBlinc* impaired leukemia cell proliferation both in vitro and in vivo. Mice transplanted with *HoxBlinc*-KO cells survived longer, had normal spleen sizes, and showed reduced leukemia cell chimerism in their bone marrow. In contrast, mice transplanted with WT cells died within a few weeks, showing signs of leukemia progression such as splenomegaly and enlarged lymph nodes. Additionally, *HoxBlinc* knockdown showed antileukemic phenotypes identical to those of the *HoxBlinc-*KO models, confirming that the effect was specifically due to *HoxBlinc* and not other DNA-regulatory elements (11).

To further understand the mechanisms for *HoxBlinc* regulation of NUP98-PHF23–mediated oncogenic homeotic gene transcription, Hamamoto and colleagues performed various genomic analyses (including ChIRP-Seq, ChIP-Seq, ATAC-Seq, and Hi-C), comparing WT and *HoxBlinc*-KO B-ALL cells. They found that *HoxBlinc* loss affected chromatin accessibility at promoter regions, leading to reduced MLL1 recruitment, decreased H3K4me3, and diminished chromatin accessibility. However, global binding of NUP98-PHF23 and CCCTC-binding factor (CTCF), a key chromatin boundary factor, remained unaffected. Importantly, loss of *HoxBlinc* disrupted the 3D genome organization, especially TAD and sub-TAD formation at Hox-associated loci (including *Hoxa*, *Hoxb*, *Meis1*, and *Kit*), without altering CTCF-driven chromatin loops, suggesting that *HoxBlinc* functions downstream of NUP98-PHF23 to regulate chromatin structure and gene expression in a CTCF-independent manner, which subsequently affects the leukemic transcriptional program (11).

Hamamoto and co-authors also showed that overexpression of *HoxBlinc* in mice resulted in hematological abnormalities resembling AML in a majority of cases, while a smaller fraction developed a B-ALL–like phenotype. These B-ALL–like mice exhibited symptoms such as splenomegaly, lymphadenopathy, leukocytosis, and severe anemia, and their B-ALL was transferable in transplantation experiments. Further examination showed that *HoxBlinc* overexpression impaired B cell development, particularly blocking progression from pro–/pre–B cell to mature B cells, both in vivo and in vitro. This developmental disruption was linked to an altered hematopoietic trajectory, favoring the myeloid and B-lymphoid lineage over others, including erythroid and basophil lineages. Single-cell RNA-Seq using HSPC subpopulations from *HoxBlinc*-transgenic mice supported the premise of a lineage bias, showing a shift toward myeloid and B cell fates, which likely contributed to the onset of both B-ALL and AML in these mice. These results underscore the potential of *HoxBlinc* to induce leukemias by altering hematopoietic differentiation pathways. Mechanistically, transgenic overexpression of *HoxBlinc* lncRNA in the hematopoietic compartment promoted leukemic TAD topology, chromatin accessibility, and homeotic oncogene transcription, resulting in leukemia in mice that was similar to NUP98-PHF23–driven leukemia (11).

Additionally, *HoxBlinc* was required for the NUP98-HOXA9 fusion oncoprotein to drive leukemogenesis. In patient samples with *NUP98-HOXA9* translocation, *HoxBlinc* expression was elevated. Silencing *HoxBlinc* using shRNA in AML cells reduced the expression of homeotic oncogenes, including *HOXA9*, *MEIS1*, *KIT*, and *JAK2*, and impaired the *NUP98-HOXA9* fusion gene signature. *HoxBlinc* depletion also affected immune processes, leukocyte activation, hematopoiesis, and transcriptional regulation. When primary NUP98-HOXA9 AML cells with reduced *HoxBlinc* were transplanted into mice, the progression of leukemia was delayed, suggesting that *HoxBlinc* was crucial for the oncogenic transformation driven by the *NUP98-HOXA9* fusion. These findings indicate that *HoxBlinc* is essential for maintaining the transcriptional profile of the HOX subset of homeotic oncogenes and necessary for the leukemogenic potential of NUP98-HOXA9 fusion proteins (11).

## Therapeutic potential of *HoxBlinc* in *NUP98* fusion–driven leukemia

The identification of *HoxBlinc* as a key player in *NUP98* fusion–driven leukemogenesis opens up potential avenues for targeted therapeutic strategies that aim to disrupt this aberrant chromatin remodeling and gene expression regulation. However, although these findings are promising, there remain several outstanding questions regarding the exact mechanisms by which *HoxBlinc* interacts with NUP98 fusions and whether other factors, such as *HOTTIP*, a lncRNA associated with the *HOXA* locus, are involved in this complex regulatory network. Furthermore, understanding how these findings translate to human leukemia and exploring the therapeutic implications of targeting *HoxBlinc* or related chromatin regulators warrant further investigation. Finally, the heterogeneity of leukemia and the interplay between different molecular drivers necessitate further study to develop more effective and universally applicable therapies.

In summary, Hamamoto et al. (11) raise an important mechanistic question as to how NUP98 fusions, particularly NUP98-PHF23, disrupt hematopoietic differentiation and contribute to leukemogenesis. Their findings highlight a role of *HoxBlinc* lncRNA in regulating *NUP98* fusion–driven leukemia, possibly by modulating chromatin structure and transcriptional programs in a CTCF-independent manner. The discovery of *HoxBlinc* lncRNA as a downstream regulator of NUP98 fusion oncoproteins offers new insights into the epigenetic mechanisms driving these malignancies and provides an exciting opportunity for future research and potential therapeutic intervention.

## Version 1 04/01/2025

Electronic publication

## References

[1] Chandra B (2022). Phase separation mediates NUP98 fusion oncoprotein leukemic transformation. Cancer Discov.

[2] Tian J (2024). The landscape of NUP98 rearrangements clinical characteristics and treatment response from 1491 acute leukemia patients. Blood Cancer J.

[3] Michmerhuizen NL (2020). Mechanistic insights and potential therapeutic approaches for NUP98-rearranged hematologic malignancies. Blood.

[4] Domingo-Reinés J (2023). The pediatric leukemia oncoprotein NUP98-KDM5A induces genomic instability that may facilitate malignant transformation. Cell Death Dis.

[5] Ealo T (2024). Cooperative insulation of regulatory domains by CTCF-dependent physical insulation and promoter competition. Nat Commun.

[6] Islam Z (2023). Active enhancers strengthen insulation by RNA-mediated CTCF binding at chromatin domain boundaries. Genome Res.

[7] Balasubramanian D (2024). Enhancer-promoter interactions can form independently of genomic distance and be functional across TAD boundaries. Nucleic Acids Res.

[8] Zhu G (2021). HOXBLINC long non-coding RNA activation promotes leukemogenesis in NPM1-mutant acute myeloid leukemia. Nat Commun.

[9] Gough SM (2011). NUP98 gene fusions and hematopoietic malignancies: common themes and new biologic insights. Blood.

[10] Xu H (2016). NUP98 Fusion Proteins Interact with the NSL and MLL1 Complexes to Drive Leukemogenesis. Cancer Cell.

[11] Hamamoto K (2025). *HoxBlinc* lncRNA reprograms CTCF-independent TADs to drive leukemic transcription and HSC dysregulation in NUP98-rearranged leukemia. J Clin Invest.

